# Psychosocial wellbeing of Berlin school children during the COVID-19 pandemic, June 2020-March 2021: Stefanie Theuring

**DOI:** 10.1093/eurpub/ckac130.018

**Published:** 2022-10-25

**Authors:** S Theuring, W van Loon, F Hommes, N Bethke, MA Mall, T Kurth, J Seybold, FP Mockenhaupt

**Affiliations:** 1 Institute of Tropical Medicine and International Health, Charité- Universitätsmedizin, Berlin, Germany; 2 Medical Directorate, Charité-Universitätsmedizin, Berlin, Germany; 3 Department of Pediatric Respiratory Medicine, Charité- Universitätsmedizin, Berlin, Germany; 4 Institute of Public Health, Charité- Universitätsmedizin, Berlin, Germany

## Abstract

**Background:**

The COVID-19 pandemic and related restrictions have affected the wellbeing of school children worldwide. Specific problems evolving during the pandemic, their extent and duration haveńt been sufficiently explored yet. We aimed at describing school childreńs psychosocial and behavioral parameters and associated factors during the COVID-19 pandemic in Berlin, Germany.

**Methods:**

Our longitudinal study included students from 24 randomly selected Berlin primary and secondary schools, assessing psychosocial wellbeing and behaviors at four time points between June 2020 and March 2021. We analyzed temporal changes in the proportions of anxiety, fear of infection, reduced health-related quality of life (HRQoL), physical activity and social contacts, as well as sociodemographic and economic factors associated with anxiety, fear of infection and HRQoL.

**Results:**

Of initially 384 recruited students, 324 still participated in the fourth study round after nine months. During the observation period, presence of anxiety symptoms increased from 26.2% (96/367) to 34.6% (62/179), and fear of infection from 28.6% (108/377) to 40.6% (73/180). The proportion of children with limited social contacts (<1/week) increased from 16.4% (61/373) to 23.5% (42/179). Low physical activity (<3 times sports/week) was consistent over time. Low HRQoL was observed among 44% (77/174). Factors associated with anxiety were female sex, increasing age, secondary school attendance, lower household income, and presence of adults with anxiety symptoms in the student's household. Fear of infection and low HRQoL were associated with anxiety.

**Conclusions:**

A substantial proportion of school children experienced unfavorable psychosocial conditions during the COVID-19 pandemic in 2020/2021. In particular, students from households with limited social and financial resilience require special attention.

**Key messages:**

- Berlin school childreńs anxiety increased significantly during the pandemic months between June 2020 and March 2021 and was associated with anxiety in the family and lower household income.
- Almost half of the schoolchildren in our study reported low health-related quality of life after the second pandemic lockdown in March 2021.

